# Mr. Kenworthy's Case of Obstruction in the Urethra

**Published:** 1809-03

**Authors:** John Kenworthy

**Affiliations:** Member of the Royal College of Surgeons, London. Dobcross, Yorkshire


### *To the Editors of the Medical and Phyſical Journal.*

Gentlemen,

As the melancholy instances of human diseases are often a source of anxiety and despondence to society at large; to the respectable body of gentlemen engaged in the alleviation of them, it is a beneficent and ineffable delight, the consciousness of ameliorating human misery. As this is a first effort at publicity, I feel on the one hand, a confidence that the liberal and benevolent lovers of science will overlook trifling errors, comparatively of no consequence; while, on the other, the malevolence of satire and ill-nature will but excite contemptuous indifference.

If you think the following (to me rather unique) case worthy a place in your useful Miscellany, I will thank you to insert it; and as the diseases of the urinary organs frequently excite considerable dispute, should it throw any beneficial light on the subject; I shall feel myself amply rewarded.

In August, 1808, I was requested to visit a man in the poor-house of Saddleworth, about 45 years of age, who was suffering excessive pain from long retention of urine. I immediately introduced the catheter, and drew away about two pints of high-coloured fœtid urine, which produced the same glutinous effect, as it dried on my hand, as a weak solution of gum arabic or glue. During the time of its flowing off, I several times applied the tip of my finger to the mouth of the catheter, for the purpose of preventing faintness by its sudden discharge. When the bladder was completely emptied, I questioned him particularly respecting the progress of his disease, and he informed me, that upwards of three years he had been troubled with uneasiness in the urinary organs, which had never during that time entirely left him; such as a frequent and ineffectual desire to make water; pain in his loins and throughout the hypogastric region, with other symptoms of disarrangement, which had gradually increased during the whole of the time. In the early stages he had no sickness, heat, nor thirst; but towards a later period, he had frequently, after using an increased exercise, or change of weather, suffered from the characteristic symptoms of sympathetic inflammatory fever, from which I suspected that some latent inflammation had been carried on; the testes were frequently painful to the touch, and particularly, when he had more uneasiness, in making water, they retracted very considerably; there was a numbness in the buttocks extending down the thighs; and within the three or four months preceding the time I first saw him, he had suffered exceedingly from nausea and vomiting; feverish symptoms and restlessness, and an excessive and frequent desire for micturition, which induced him to strain so violently at times, as to force the rectum down to the extent of several inches. He informed me, that in June he had been examined by a Mr. B. who sounded him, and gave it as his decided opinion, there was a calculus in the vesica urinaria; he accordingly ordered him to Manchester Infirmary for operation, the man not being capable of discharging the expences likely to be incurred; but as the surgeons of that institution could not possibly discover any thing of the nature of a stone, he was consequently returned without operation.

While yet the catheter was retained within the urethra, I endeavoured to examine whether there was any extraneous body lodged within the vesica urinaria, and in passing the point of the instrument carefully in different directions, I discovered a tumour of considerable magnitude, attached near to the neck of the bladder, of a rather soft consistence, yielding to the point of the catheter, without inducing the least pain. The cause of retention was evidently mechanically effected by this preternatural growth, acting as a valve. Pursuing my examination, as to the bulk, form, &c. of the tumour, I accidentally ruptured one side of it, and a large quantity of hydatids, from three-fourths of an inch diameter to the size of a pin's head, were slowly discharged through the canal of the urethra, perhaps, to the amount of three half pints. I ordered him the following mixture:

℞. Ol. amygdal dulc. Mucil. g. arab. aa. ʒvj. Tinct. opii gtta. xxx. Aquæ distillat. ℥ivß. M. signa. Capt. cochl. major. ij. 3 qq. hora.

The pubes and perinæum to be fomented with warm water; confined him to a recumbent position, and directed the use of infus. simenis lini, together with a diet of milk, broths, &c. He continued in this course about a fortnight; he has suffered no material inconvenience since, and now enjoys a good state of health, except that at times he feels a slight uneasiness from excess of exercise, or the variations of the atmosphere.

I am, &c.

JOHN KENWORTHY,

Member of the Royal College of Surgeons, London.

*Dobcross, Yorkshire, Feb.* 6, 1809.

